# Microbial Natural Products as Potential Inhibitors of SARS-CoV-2 Main Protease (M^pro^)

**DOI:** 10.3390/microorganisms8070970

**Published:** 2020-06-29

**Authors:** Ahmed M. Sayed, Hani A. Alhadrami, Ahmed O. El-Gendy, Yara I. Shamikh, Lassaad Belbahri, Hossam M. Hassan, Usama Ramadan Abdelmohsen, Mostafa E. Rateb

**Affiliations:** 1 Department of Pharmacognosy, Faculty of Pharmacy, Nahda University, 62513 Beni-Suef, Egypt; ahmedpharma8530@gmail.com; 2 Department of Medical Laboratory Technology, Faculty of Applied Medical Sciences, King Abdulaziz University, Jeddah 21589, Saudi Arabia; hanialhadrami@kau.edu.sa; 3 Special Infectious Agent Unit, King Fahd Medical Research Centre, King Abdulaziz University, Jeddah 21589, Saudi Arabia; 4 Department of Microbiology, Faculty of Pharmacy, Beni-Suef University, 62514Beni-Suef, Egypt; ahmed.elgendy@pharm.bsu.edu.eg; 5 Department of Microbiology & Immunology, Nahda University, Beni-Suef 62513, Egypt; yara.shamikh@nub.edu.eg; 6 Department of Virology, Egypt Center for Research and Regenerative Medicine (ECRRM), 11517 Cairo, Egypt; 7 Laboratory of Soil Biology, Department of Biology, University of Neuchatel, 2000 Neuchatel, Switzerland; lassaad.belbahri@unine.ch; 8 Department of Pharmacognosy, Faculty of Pharmacy, Beni-Suef University, 62514 Beni-Suef, Egypt; abuh20050@yahoo.com; 9 Department of Pharmacognosy, Faculty of Pharmacy, Minia University, 61519 Minia, Egypt; 10 Department of Pharmacognosy, Faculty of Pharmacy, Deraya University, 61111 New Minia, Egypt; 11 School of Computing, Engineering & Physical Sciences, University of the West of Scotland, Paisley PA1 2BE, UK

**Keywords:** SARS-CoV-2, Covid-19, M^pro^, microbial natural products, docking, molecular dynamic simulation

## Abstract

The main protease (M^pro^) of the newly emerged severe acute respiratory syndrome coronavirus 2 (SARS-CoV-2) was subjected to hyphenated pharmacophoric-based and structural-based virtual screenings using a library of microbial natural products (>24,000 compounds). Subsequent filtering of the resulted hits according to the Lipinski’s rules was applied to select only the drug-like molecules. Top-scoring hits were further filtered out depending on their ability to show constant good binding affinities towards the molecular dynamic simulation (MDS)-derived enzyme’s conformers. Final MDS experiments were performed on the ligand–protein complexes (compounds **1**–**12**, Table S1) to verify their binding modes and calculate their binding free energy. Consequently, a final selection of six compounds (**1**–**6**) was proposed to possess high potential as anti-SARS-CoV-2 drug candidates. Our study provides insight into the role of the M^pro^ structural flexibility during interactions with the possible inhibitors and sheds light on the structure-based design of anti-coronavirus disease 2019 (COVID-19) therapeutics targeting SARS-CoV-2.

## 1. Introduction

In 2002, the first spread of coronavirus associated with severe acute respiratory syndrome coronavirus (SARS-CoV) emerged in southern China. This outbreak successfully subsided by the summer of 2003 [1], with no more than 8500 confirmed infections and just over 900 deaths worldwide [2]. Upon the spread of this outbreak, the global response was immediate to characterize its causative agent as a novel coronavirus (SARS-CoV) [3]. The recurrence of SARS in the Guangdong province of China in December 2003 [4] illustrated the need to continue efforts to study this virus and its key molecular targets to develop appropriate therapeutics for its treatment. In 2012, another coronavirus wave originating in Jeddah, Saudi Arabia emerged and spread within and beyond the Middle East. The reported strain (MERS-CoV) was associated with severe pneumonia and multi-organ failure [5]. The limited number of infected cases during the previous coronaviruses waves did not encourage a serious worldwide development of effective treatments.

Recently and until June 2020, the outbreak of a new coronavirus (SARS-CoV-2) that originated from Wuhan, China, in late 2019, has led to more than 9.3 million infections and more than 479,000 deaths throughout 216 countries without any proven antiviral agents or effective vaccine according to WHO official updates. However, repurposing of previous medications has shown some reported clinical improvements [6]. This time, worldwide efforts are being made to characterize molecular targets, pivotal for the development of anti-coronavirus therapies. The term coronavirus was coined according to its corona-like appearance in the electron microscope, due to its spikes that radiate outwards from the viral envelope. The spherical capsid envelops a positive-strand RNA genome of about 30 kb, which is considered the largest of its kind. The viral genome is predominated by two open reading frames that are connected by a ribosomal frameshift site and encode the two replicase proteins, pp1a and pp1ab [7]. These polyproteins are cleaved by the viral main protease (M^pro)^, also called chymotrypsin-like protease, 3CL^Pro^ [8,9]. M^pro^ is considered a highly conserved molecular target across coronaviruses, and hence, it was designated as a potential target for anti-coronavirus drug development [10]. Earlier reports on viral protease inhibitors (i.e., the HIV protease inhibitor lopinavir) revealed significant in vitro anti-SARS-CoV-2 activities [11].

Natural products along with natural product-inspired synthetic and semisynthetic compounds are still an excellent structural motif for the discovery of new therapeutics, including antiviral agents. Natural products derived from microbial sources are considered unique in their chemical diversity in comparison with plant-derived ones. Approximately 53% of the FDA-approved natural products-based drugs are of microbial origin, notably the antiviral ones [12]. For example, Ara-A (**9**) (also known as vidarabine) is considered one of the earliest antiviral nucleoside analogues that was reported from *Streptomyces antibioticus* [13]. Afterwards, several nucleoside-based antiviral agents of microbial origin were developed [14]. Additionally, several ansamycins-based antibiotics (e.g., rifamycin, **10**) have shown interesting antiviral properties against a wide range of infectious viruses [15]. Furthermore, the well-known immunomodulatory drug of fungal origin mycophenolic acid (**11**) has also shown a broad antiviral activity [15]

**Figure 1:**
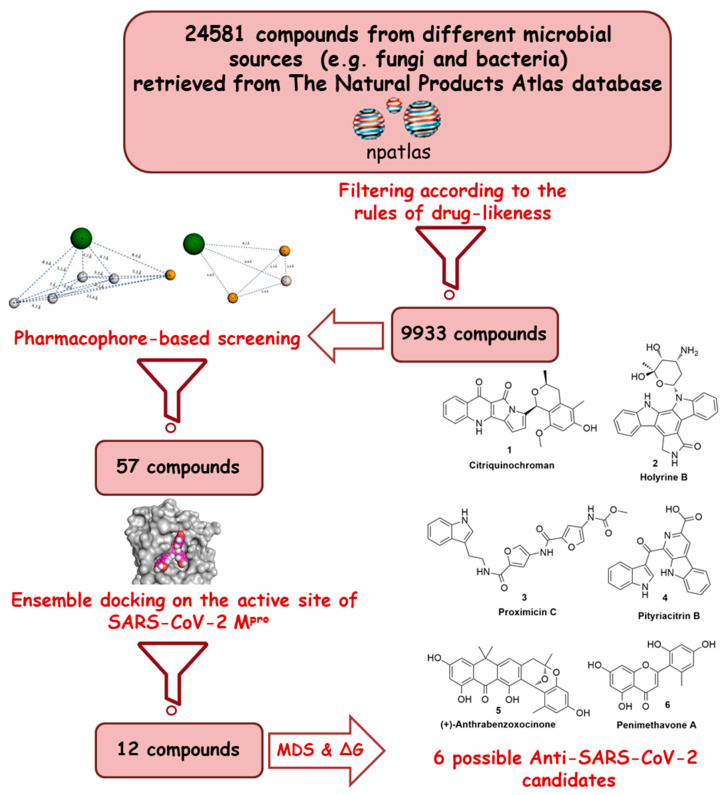
Applied strategy in the present study.

Recently, the microbial-derived FDA-approved anti-parasitic drug ivermectin (**12**), a semisynthetic pentacyclic sixteen-membered lactone derived from the soil bacterium *Streptomyces avermitilis*, proved to be an effective in vitro inhibitor of SARS-CoV-2 replication [16,17]. In this context, several in silico techniques that have recently gained a lot of attention in drug discovery campaigns (e.g., structure and ligand-based virtual screening, docking and molecular dynamics) [18,19]. Hence, we initiated a virtual screening of a big library of microbial natural products (more than 24,000 compounds) aimed at the discovery of potential drug candidates against the SARS-CoV-2 M^pro^, taking into account the drug-likeness properties to select only druggable candidates. Simple docking protocols do not take into consideration the flexible nature of proteins; therefore, their success rates in most cases are between 60–70% [20]. To increase the docking performance, top hits retrieved from the primary pharmacophore-based screening were further docked on a series of receptor conformers (i.e., ensemble docking) [21] taken from the molecular dynamic simulation (MDS) to consider the factor of the binding pockets’ flexibility. Finally, further MDS of the protein–ligand complexes were performed to verify our docking experiments and calculate their binding free energy (ΔG). Drug candidates proposed in this study could provide a promising starting point for the in vitro and in vivo testing and further development of potential drug leads against the newly emerged coronavirus disease 2019 (COVID-19). The procedure of the current investigation is depicted in Figure 1.

## 2. Materials and Methods

### 2.1. Preparation of SARS-CoV-2 M^pro^ and the Compounds Dataset

Crystal structures of M^pro^ (PDB code: 6LU7 and 6M2N) were obtained from the Protein Data Bank (http://www.pdb.org), and all heteroatoms and water molecules were removed for MDS and molecular docking studies. The chemical structures of the tested microbial specialized metabolites were retrieved from the online dataset; The Natural Products Atlas (https://www.npatlas.org/joomla/index.php) [22] with a final compiled dataset consisting of 24,581 compounds. Subsequently, this library of compounds was subjected to LigandScout software [23] to select 9933 compounds that showed drug-like properties (i.e., obey Lipinski’s role of five) [24].

### 2.2. Molecular Dynamic Simulation

Molecular dynamic simulations (MDS) for the free M^pro^ enzyme and ligand–enzyme complexes were performed using the Nanoscale Molecular Dynamics (NAMD) 2.6 software [25], employing the CHARMM27 force field [26]. Hydrogen atoms were added to initial coordinates for M^pro^ using the psfgen plugin included in the Visual Molecular Dynamic (VMD) 1.9 software [27]. Subsequently, the protein system was solvated using TIP3P water particles and 0.15 M NaCl. The equilibration procedure comprised 1500 minimization steps followed by 30 ps of MDS at 10 k with fixed protein atoms. Then, the entire system was minimized over 1500 steps at 0 K, followed by gradual heating from 10 to 310 K using temperature reassignment during the initial 60 ps of the 100 ps equilibration MDS. The final step involved NTP simulation (30 ps) using the Nose–Hoover Langevin piston pressure control at 310 K and 1.01325 bars for density (volume) fitting [28]. Thereafter, the MDS was continued for 25 ns for the entire system (20 ns for the enzyme–ligand complexes). The trajectory was stored every 0.1 ns and further analyzed with the VMD 1.9 software. The MDS output over 25 ns provided several structural conformers that were sampled every 0.1 ns to evaluate the conformational changes of the entire protein structure to analyze the root mean square deviation (RMSD) and root mean square fluctuation (RMSF). All parameters and topologies of the compounds selected for MDS (1–12, Table S1) were prepared using the online software Ligand Reader & Modeler (http://www.charmm-gui.org/?doc=input/ligandrm) [29] and the VMD Force Field Toolkit (ffTK) [27]. Binding free energy calculations (Δ*G*) were performed using the free energy perturbation (FEB) method through the web-based software Absolute Ligand Binder [30] together with the MDS software NAMD 2.6 [25]. Additionally, they were calculated using another web-based software, namely *K*_DEEP_ (https://www.playmolecule.org/Kdeep/), which applies a neural-networking algorithm during its computations [31].

### 2.3. Pharmacophore-Based Virtual Screening and Molecular Docking

The pharmacophore-based screening was performed by the online sever Pharmit (http://pharmit.csb.pitt.edu/) [32]. The pharmacophore models were constructed from the SARS-M^pro^ enzymes co-crystallized with baicalein (7) and N3 (8). All the pre-installed Pharmit parameters remained unchanged. The resulting two models were used for the virtual screening of the compounds filtered from the prepared microbial natural products library (9933 compounds). Afterwards, compounds with RMSD > 2Å were excluded. Docking experiments were performed using AutoDock Vina software [33]. All compounds that fitted into the predetermined pharmacophore models (57 compounds) were then subjected to molecular docking against the M^pro^’s active site conformers that were sampled from the MDS every 5 ns (i.e., ensemble docking). We set an average docking score of −10 kcal/mol (the average docking score of compound **7**) as a cut-off to select the top-scoring hits. Afterwards, the retrieved top hits (1–12, Table S1) were ranked according to their binding energies (Table 1 and Table S1). The generated docking poses were visualized and analyzed using Pymol software [34].

## 3. Results and Discussion

### 3.1. Structure and Dynamics of the SARS-CoV-2 M^pro^

**Figure 2:**
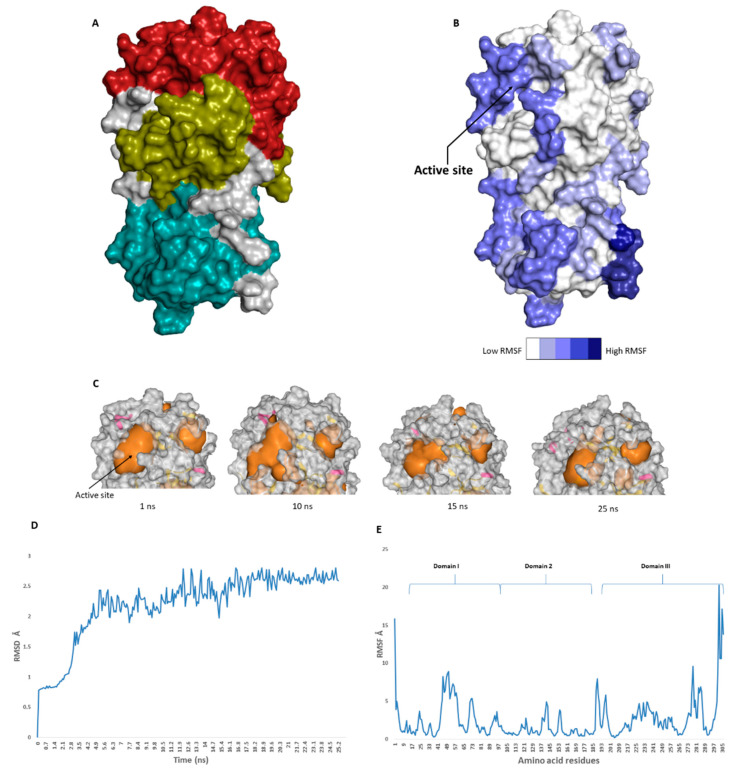
(**A**): The main domains (M^pro^) in severe acute respiratory syndrome coronavirus 2 (SARS-CoV-2) (Brick red: Domain I, Golden yellow: Domain II, Cyan: Domain III), (**B**): Heat-map illustrating the flexible regions on SARS-CoV-2 M^pro^, (**C**): The active site volume changes during the course of molecular dynamic simulation (MDS) (**D** and **E**): root main square deviation (RMSD) and root main square fluctuation (RMSF) of SARS-CoV-2 M^pro^ after 25 ns of MDS.

The catalytic site of the SARS-CoV-2 M^pro^ was found to be the largest cavity on the whole protein (static volume = 385.56 Å^3^) and is located in domains I and II (residues 11 to 99 and 100 to 182, respectively, Figure 2A). Additionally, it is smaller than the earlier SARS-CoV M^pro^ (static volume = 447.7 Å^3^) [35]. It is worth noting that the enzyme’s domain III, which consists of a globular cluster of five helices, is present only in coronaviruses and is responsible for the regulation of M^pro^ dimerization [36]. MDS of the reported SARS-CoV-2 M^pro^ (PDB: 6LU7) [10] was performed to study the conformational changes in the active site using clustering analysis (conformation was sampled every 0.1 ns). The carbon alpha (Cα) root main square deviation (RMSD) values with respect to the initial structure were calculated for 25 ns of the simulation and were found to oscillate from 0.78 to 2.78 Å with a median value of (2.51 Å), reaching equilibrium (i.e., plateau) at 4.8 ns (Figure 2D). Regarding root mean square fluctuation (RMSF) values, they demonstrated that M^pro^ had moderate flexibility (average RMSF = 2.43 Å, Figure 2E), where the active site showed the highest conformational changes (average RMSF = 3.1 Å), particularly at the THR-45 to ILE-59 loop (Figure 2B), which showed high fluctuations (i.e., RMSF values ranged from 5.2 to 8.9 Å, Figure 2E).

The volume of the active site was 399.56 Å^3^ at the beginning of the simulation (Figure 2C), and during the MDS, it gradually increased to reach 479.65 Å^3^ at 10 ns, and then began to decrease to reach 314.63 Å^3^ at the end of the MDS. Docking and virtual screening studies on such flexible catalytic active sites using only their crystalized static form would lead to poor prediction results. Hence, considering multiple structural conformations (i.e., ensemble docking) derived from the MDS study for our virtual screening in the present investigation could significantly improve the predicted results.

### 3.2. Pharmacophore-Based Modeling and Screening

To discover potential naturally occurring ligands that could block the M^pro^ active site, an extensive specialized microbial natural product database (The Natural Product Atlas) containing more than 24,000 different compounds was utilized for. Firstly, the database was filtered according to drug-likeness (Lipinski’s rules [24]) to get 9933 drug-like candidates.

**Figure 3:**
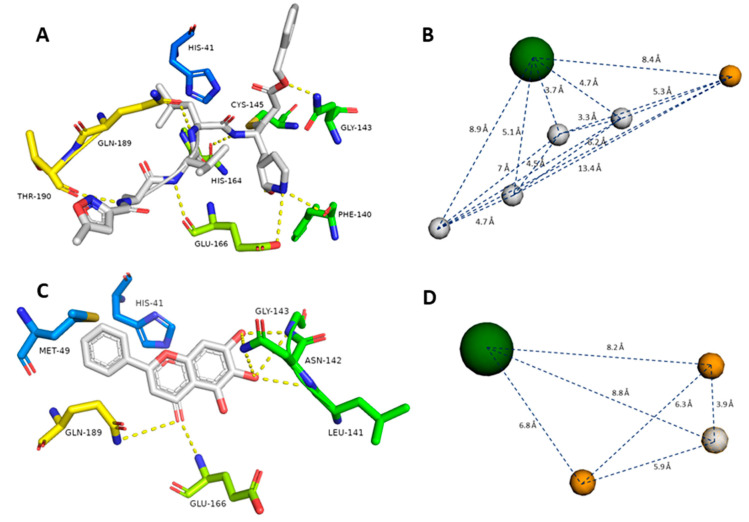
Generated pharmacophore models (**B** and **D**) according to the previously reported co-crystalized ligands (**7** and **8**, **A** and **C**). Gray spheres indicate hydrogen bond donors, orange spheres indicate hydrogen bond acceptors, and green spheres indicate hydrophobic centers. Green amino acid residues represent the S1 pocket, blue amino acid residues represent the S2 pocket, yellow amino acid residues represent the S3 and S4 pockets in the M^pro^ active site (**A** and **C**).

The crystal structure of SARS-CoV-2 M^pro^ (PDB id: 6LU7) was reported earlier, along with its co-crystallized peptide inhibitor N3 [10]. N3 (**8**) is fitted inside the M^pro^ active site through multiple strong H-bonds (e.g., GLY-143, HIS-164, GLU-166, GLN-189, and THR-190) alongside a covalent bond with CYS-145. Additionally, its isopropyl group is imbedded inside a hydrophobic pocket that consists of HIS-41, MET-49, and GLN-189 (Figure 3A, Table 1).

Recently, a novel flavonoid-based non-covalent inhibitor (baicalein, **7**) (PDB code: 6M2N) was found to accept seven H-bonds from LEU-141, ASN-142, GLY-143, Glu-166, and GLN-189. Moreover, its phenyl moiety was also fitted inside the hydrophobic pocket of HIS-41, MET-49, and GLN-189 (Figure 3C, Table 1). The predetermination of pharmacophoric characteristics prior to structure-based virtual screening would help in selecting the best hits with the best interaction inside the binding pocket [37].

To define the essential features of the interaction inside the M^pro^’s active site [32], two structure-based pharmacophore models were constructed depending on the two previously described inhibitors. The N3 (**8**)-based-pharmacophore model had the following features: four H-bond donors derived from four amide groups, one carboxyl group-derived oxygen atom as an H-bond acceptor, and the isopropyl group to represent a hydrophobic center (Figure 3B). On the other hand, the baicalein (**7**)-based pharmacophore model showed the following features: two H-bond acceptors derived from one hydroxyl group and the ketonic oxygen, one H-bond donor derived from another hydroxyl group, and the phenyl group to represent a hydrophobic center (Figure 3D).

Subsequently, these binding site-derived pharmacophore models were used in our virtual screening against the MND-selected compounds (the 9933 compounds that obey Lipinski’s rules) using the online server Pharmit [32]. This allowed us to select the compounds with predetermined pharmacophoric features capable of interacting with the reported key residues. This filtration step led to the recognition of 363 compounds that met the predetermined model features, of which we selected only 57 compounds (i.e., showed RMSD values lower than 2 Å with respect to the co-crystalized ligands **7** and **8**) to undergo a subsequent docking-based virtual screening.

### 3.3. Molecular Docking and Binding Mode Investigation

**Figure 4:**
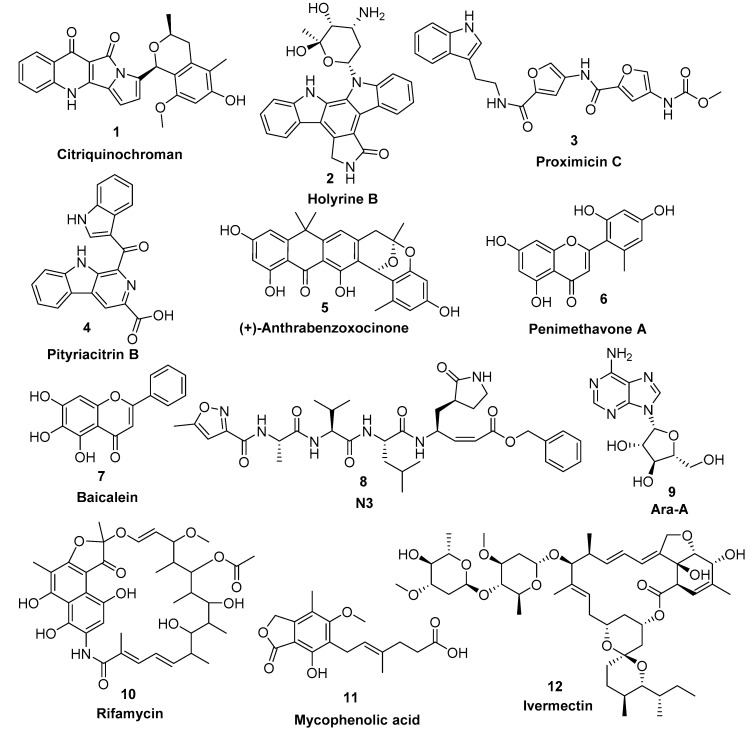
Top-scoring compounds (**1**–**6**) retrieved from the in silico virtual screening on the M^pro^ active site along with the co-crystallized inhibitors **7** and **8** in addition to the previously reported antiviral microbial natural products (**9–12**).

Docking of the selected compounds inside the M^pro^ active site was performed on AutoDock Vina, which was able to reproduce the binding mode of the co-crystalized ligands [37], N3 (**8**) and baicalein (**7**), with an RMSD values of 1.22 and 0.51 Å, respectively. Fifty seven compounds were filtered according to the predetermined pharmacophoric features and drug-likeness properties, and thereafter docked separately on the SARS-CoV-2 M^pro^ active site using several snapshots (every 5 ns) derived from the MDS (i.e., ensemble docking). This allowed us to further select the best binding compounds taking into consideration the flexibility of the active site. Top-scoring hits (those with an average binding energy score > −10 kcal/mol, Table S1 and Figure S1) with binding modes comparable with both N3 (**8**) and baicalein (7), were then subjected to MDS and binding free energy computation to further verify the suggested pharmacophore models, docking-derived binding poses, and binding affinities. Only compounds **1**–**6** (Figure 4 and Table 1) exhibited stable binding orientations inside the enzyme binding pocket throughout the MDS (Figures S2–S13) and constant binding energies higher than that of the co-crystallized inhibitors, i.e., compounds **7** and **8**.

**Figure 5:**
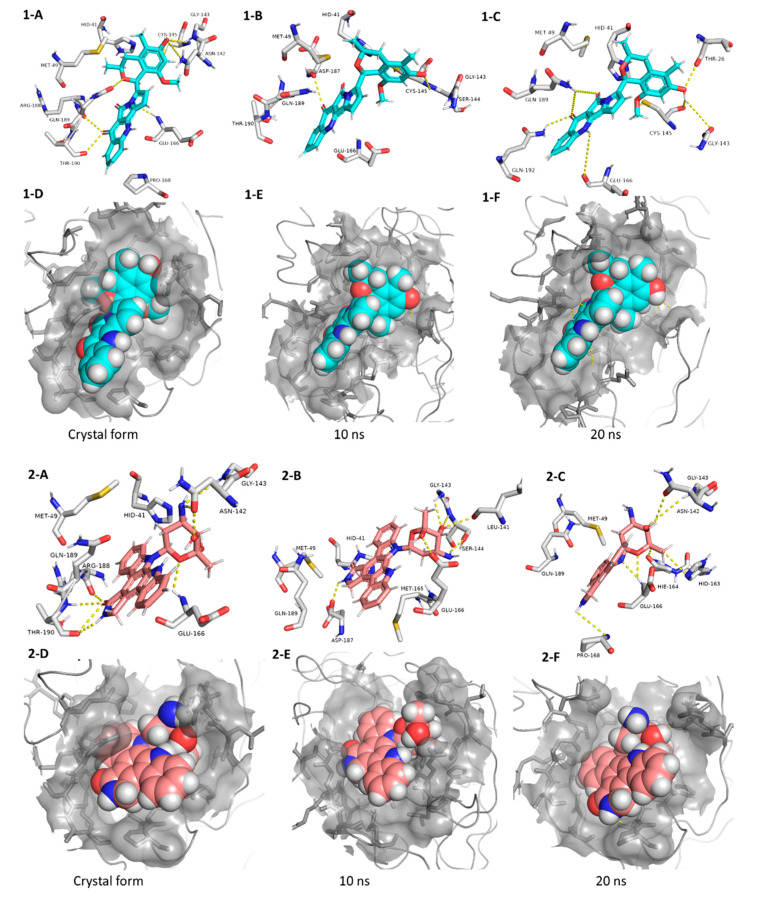
Interactions and binding modes of compounds **1** and **2** (Blue and red molecules, respectively) inside the M^pro^ active site in the crystal form and during MDS (**1-A**–**2-F**).

Our M^pro^ top-scoring ligands indicated that the best hit was citriquinochroman (**1**). This N-containing polyketide was first isolated from the endophytic fungus *Penicillium citrinum* in 2013 and showed moderate anticancer activity [38]. It showed the least binding energy (ΔG_average_ = −12.4 kcal/ mol), with perfect fitting inside the enzyme active site in the crystallized form, where it anchored itself via a network of H-bond interactions with the reported key binding residues (7 H-bonds) [10]. Despite the flexibility of the enzyme active site, citriquinochroman (**1**) was able to keep its orientation during the course of MDS (Figure 5 and Figure S2) with a transient drop in its binding affinity at 3–5.5 ns (ΔG_Vina_ = −8.9 kcal/mol). Afterwards, both THR-26 and GLN-192 stabilized compound **1** with additional H-bond interactions until the end of the MDS (Figure 5).

The second most promising hit was holyrine B (**2**), an indolocarbazole alkaloid that was previously isolated from a marine-derived actinomycete [39]. Holyrine B (**2**) exhibited binding modes similar to that of citriquinochroman (**1**), where it also interacted through H-bonding or hydrophobic interactions with the reported key amino acid residues [10]. Additionally, it was able to keep these strong interactions throughout the MDS and form additional H-bonds and hydrophobic interactions with extra amino acid residues like HID-163, HIE-164, PRO-168, and MET-165 (Figure 5 and Table 1).

**Figure 6:**
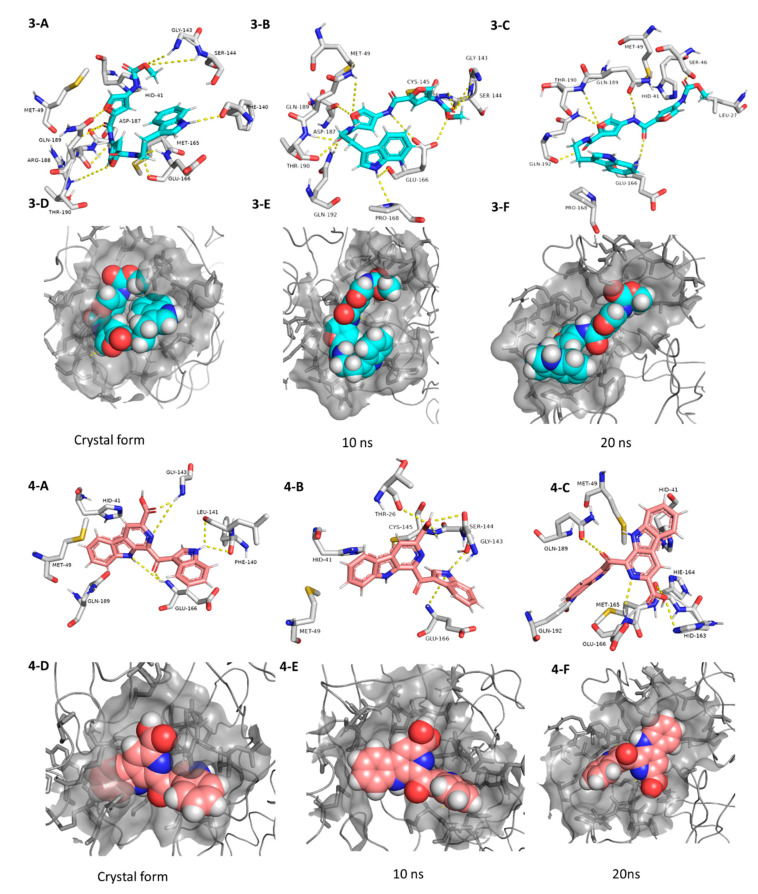
Interactions and binding modes of compounds **3** and **4** (Blue and red molecules, respectively) inside the M^pro^ active site in the crystal form and during MDS (**3-A**–**4-F**).

The third best hit was the aminofuran antibiotic proximicin C (**3**), which was isolated from the marine actinomycete *Verrucosispora* MG-37 [40]. Proximicin C (**3**) showed an interesting binding pose inside the enzyme active site in the crystalized form where it interacted with several amino acid residues including the reported ones (9 H-bonds and 2 hydrophobic interactions, Figure 6). During the MDS and in contrast to the previous candidates (**1** and **2**), proximicin C’s (**3**) binding affinity remained constant for 5 ns and started to increase afterwards until the end of the simulation. Such stable fitting inside the binding site could be attributed to the molecular flexibility of this compound that enabled it to accommodate itself well inside the changing active site of SARS-CoV-2 M^pro^ (Figure 6).

Pityriacitrin B (**4**) showed excellent fitting inside the M^pro^ active site, where the phenyl moiety of the β-carboline part was impeded inside the hydrophobic pocket (HID-41, MET-94, GLN-189), while the other indole arm together with the β-carboline’s pyridine moiety interacted with the key binding amino acid residues (PHE-140, LEU-141, GLY-143, and GLU-166, Figure 6). Moreover, it was able to not only keep these interactions throughout the MDS, but also to form additional strong H-bonds with THR-26, SER-144, and CYS-145. Pityriacitrin B (**4**) was first isolated from the human pathogenic yeast *Malassezia furfur* [41]. Later on, it was identified as an efficient UV absorbing agent [42].

**Figure 7:**
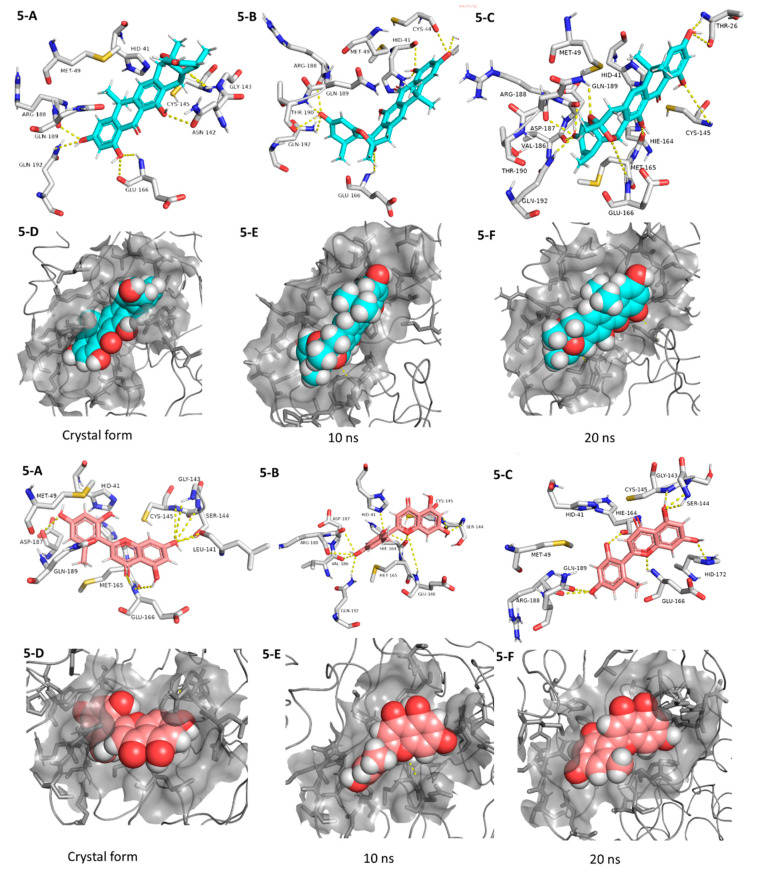
Interactions and binding modes of compounds **5** and **6** (Blue and red molecules, respectively) inside the M^pro^ active site in the crystal form and during MDS (**5-A**–**6-F**).

Coming to our next hit, (+)-anthrabenzoxocinone (**5**), it showed an interaction pattern similar to the previous candidates (**1**–**4**, and **7**, **8**) inside the crystallized form of the enzyme active site. However, from the beginning of the MDS, this compound gradually detached itself from the binding site, and starting from 7.2 ns, it took a different orientation with better interactions (Figure 7 and Table 1). At the end of the MDS, (+)-anthrabenzoxocinone (**5**) was able to form a wide network of H-bonds (10 H-bonds) and hydrophobic interactions (4 hydrophobic interactions) (Figure 7). Anthrabenzoxocinone (**5**) was isolated from a soil-derived *Streptomyces* sp. in 2014 [43].

Finally, penimethavone A (**6**), which has a flavone structure similar to the co-crystalized ligand (baicalein, **7**), was able to form molecular interactions almost identical to those of the co-crystalized ligand (**7**) (Figure 3C and Figure 7, Table 1). Additionally, it adopted a stable binding mode during the MDS, particularly near the end of simulation when it expanded its H-bonds network to involve extra binding residues like HIE-164 and HID-172. Penimethavone A (**6**) is an unusual flavone derivative with a methylated B-ring that was isolated from the gorgonian marine soft coral-derived *Penicillium chrysogenum* [44].

## 4. Conclusions

The SARS-CoV-2 pandemic crisis has inspired scientists with diverse backgrounds to help with a speedy discovery of potential treatments or vaccines. In the present virtual screening and molecular modelling study, we suggested that the active site of the newly emerged SARS-CoV-2 M^pro^ is quite flexible. Thus, its utilization in just simple docking experiments could lead to inaccurate results. Consequently, this catalytic active site was utilized in a combination of ligand-based followed by structural-based virtual screening against a big library of microbial-derived specialized metabolites, which was initially filtered according to the drug-likeness of its molecules. Top-scoring hits were further subjected to an ensemble docking protocol depending on the enzyme-generated conformers during the MDS. This step allowed us to select only ligands with stable binding affinity and modes for considering the flexibility of the active binding site. MDS, together with binding energy and affinity computations were performed for the selected hits as a final validation step to nominate six molecules with possible high potential to modulate/inhibit the SARS-CoV-2 M^pro^ active site. This study emphasized the power of computer-aided drug design and modelling in speeding up the process of drug discovery, which is currently an urgent need under the spread of COVID-19. It also highlighted the ability of natural products, particularly those of high structural diversity like microbial-derived metabolites, to provide potential drug-leads. Further in vitro testing of the drug candidates retrieved from our study is highly recommended as a promising starting point for the rapid development of drug leads against newly emerged COVID-19.

## Figures and Tables

**Table 1:** M^pro^ top-scoring ligands alongside their binding energies using different calculation methods and their molecular interactions inside the active site.

Ligand	ΔG_Vina_ (kcal/mol)	ΔG * _FEP_ (kcal/mol)	ΔG ** *K*_DEEP_ (kcal/mol)	ΔG_average_ (kcal/mol)	Hydrogen Bonding Interactions	Hydrophobic Interactions
Citriquinochroman (1)	−14.7	−11.9	−10.5	−12.4	THR-26, ASN-142, GLY-143, CYS-145, GLU-166, ASP-187, ARG-188, GLN-189, THR-190, GLN-192	HID-41, MET-49, PRO-168
Holyrine B (2)	−14.5	−11.5	−10.9	−12.3	LEU-141, ASN-142, GLY-143, SER-144, CYS-145, HID-163, HIE-164, GLU-166, PRO-168, ASP-187, ARG-188, GLN-189, THR-190, GLN-192	HID-41, MET-49, MET-165, PRO-168
Proximicin C (3)	−14.1	−12.1	−10.3	−12.2	GLY-143, SER-144, CYS-145, GLU-166, PRO-168, ASP-187, ARG-188, GLN-189, THR-190	Leu-27, HID-41, MET-49, MET-165, PRO-168
Pityriacitrin B (4)	−13.4	−12.1	−11.1	−12.2	PHE-140, LEU-141, GLY-143, SER-144, CYS-145, HID-163, HIE-164, MET-165, GLU-166, GLN-189	HID-41, MET-49, GLN-189
Anthrabenzoxocinone (5)	−13.2	−10.3	−9.5	−11	THR-26, HID-41, CYS-44, ASN-142, GLY-143, CYS-145, HIE-164, HIE-164, MET-165, GLU-166, VAL-186, ASP-187, ARG-188, GLN-189, THR-190, GLN-192	HID-41, MET-49, MET-165, GLN-189
Penimethavone A (6)	−12.1	−11.4	−8.9	−10.8	LEU-141, GLY-143, SER-144, CYS-145, HIE-164, HIE-164, MET-165, GLU-166, HID-172, VAL-186, ASP-187, ARG-188, GLN-189, GLN-192	HID-41, MET-49, MET-165, GLN-189
Co-crystalized ligand (7)	−10.1	−9.2	−8.9	−9.4	LEU-141, ASN-142, GLY-143, GLU-166, GLN-189.	HID-41, MET-49, GLN-189
Co-crystalized ligand (8)	−10.9	−11.4	−9.4	−10.6	PHE-140, GLY-143, CYS-145, HIE-164, GLU-166, GLN-189, THR-190.	HID-41, MET-49, GLN-189

* Binding free energy calculated by the free energy perturbation (FEB) method [30], ** Binding free energy calculated by a neural networking method (*K*_DEEP_) [31].

## Supplementary Materials

The following are available online at https://www.mdpi.com/2076-2607/8/7/970/s1, Figure S1: Structures of the top-scoring compounds, Figures S2–S13: RMSDs of the M^pro^ enzyme-ligand complexes and the docking binding poses of compounds (**1**–**12**) inside the binding pocket of the M^pro^’s enzyme, Table S1: Top-scoring hits retrieved from the structural-based virtual screening.
