# Supplementary material for: Microbial Natural Products as Potential Inhibitors of SARS-CoV-2 Main Protease (Mpro)
Source: Microorganisms. 2020 Jun 29;8(7):970. doi: 10.3390/microorganisms8070970 (PMC7409236; doi:10.3390/microorganisms8070970)
Supplement: Supplementary file 1 [file microorganisms-08-00970-s001.pdf]

Supplemental Material

Microbial Natural Products as Potential Inhibitors of SARS-CoV-2 Main Protease (M^{pro})

Ahmed M. Sayed [1,†], Hani A. Alhadrami [2,3,†], Ahmed O. El-Gendy [4], Yara I. Shamikh [5,6], Lassaad Belbahri [7], Hossam M. Hassan [8], Usama Ramadan Abdelmohsen [9,10,*] and Mostafa E. Rateb [11,*]

[1] Department of Pharmacognosy, Faculty of Pharmacy, Nahda University, 62513 Beni-Suef, Egypt
[2] Department of Medical Laboratory Technology, Faculty of Applied Medical Sciences, King Abdulaziz University, Jeddah 21589, Saudi Arabia
[3] Special Infectious Agent Unit, King Fahd Medical Research Centre, King Abdulaziz University, Jeddah 21589, Saudi Arabia
[4] Department of Microbiology, Faculty of Pharmacy, Beni-Suef University, 62514Beni-Suef, Egypt
[5] Department of Microbiology & Immunology, Nahda University, Beni-Suef 62513, Egypt
[6] Department of Virology, Egypt Center for Research and Regenerative Medicine (ECRRM), 11517 Cairo, Egypt
[7] Laboratory of Soil Biology, Department of Biology, University of Neuchatel, 2000 Neuchatel, Switzerland
[8] Department of Pharmacognosy, Faculty of Pharmacy, Beni-Suef University, 62514 Beni-Suef, Egypt
[9] Department of Pharmacognosy, Faculty of Pharmacy, Minia University, 61519 Minia, Egypt
[10] Department of Pharmacognosy, Faculty of Pharmacy, Deraya University, 61111 New Minia, Egypt
[11] School of Computing, Engineering & Physical Sciences, University of the West of Scotland, Paisley PA1 2BE, UK
[*] Correspondence: usama.ramadan@mu.edu.eg (U.R.A.); Mostafa.Rateb@uws.ac.uk (M.E.R.)
[†] These authors contributed equally to this work

Table S1. Top-scoring hits retrieved from the structural-based virtual screening.

No.	Name	ΔG Vina (kcal/mol)	ΔG*$_{FEP}$ (kcal/mol)	ΔG**$_{KDEEP}$ (kcal/mol)	ΔG average (kcal/mol)
1	Citriquinochroman	-14.7	-11.9	-10.5	-12.4
2	Holyrine B	-14.5	-11.5	-10.9	-12.3
3	Proximicin C	-14.1	-12.1	-10.3	-12.2
4	Pityriacitrin B	-13.4	-12.1	-11.1	-12.2
5	Anthrabenzoxocinone	-13.2	-10.3	-9.5	-11
6	Penimethavone A	-12.1	-11.4	-8.9	-10.8
7	JBIR-90	-11.5	-7.2	-8.9	-9.2
8	Xanthoradone A	-11.4	-6.5	-9.1	-9
9	Siphonazole B	-11.1	-6.1	-8.8	-8.7
10	Piperitol	-10.9	-6.4	-8.4	-8.6
11	Endophenazine D	-10.7	-5.8	-8.3	-8.3
12	Ammonificin C	-10.4	-5.5	-8.1	-8

*Binding free energy calculated by FEB method. **Binding free energy calculated by a neural networking method (*K*DEEP).

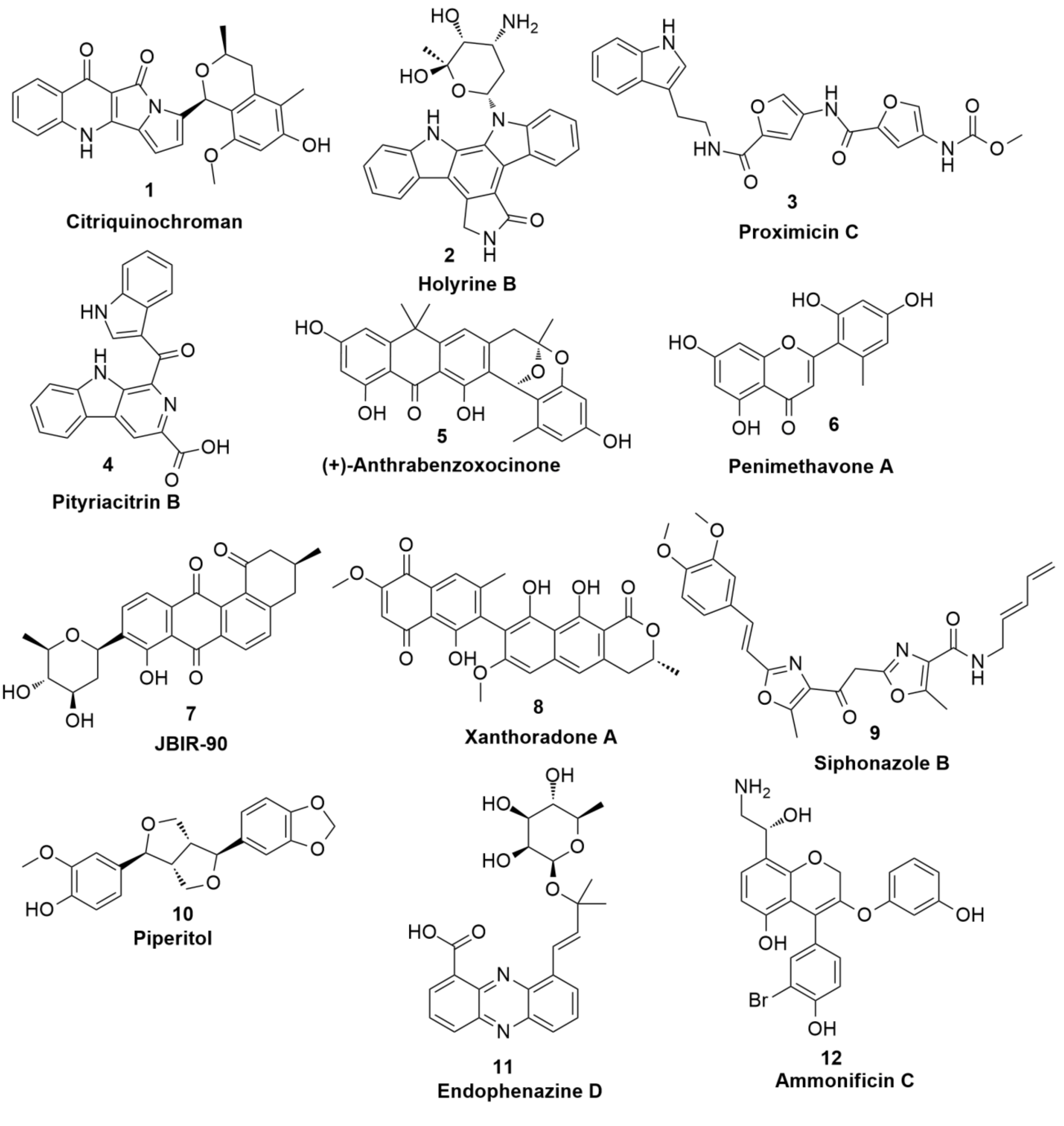

Figure S1. Structures of top-scoring compounds.

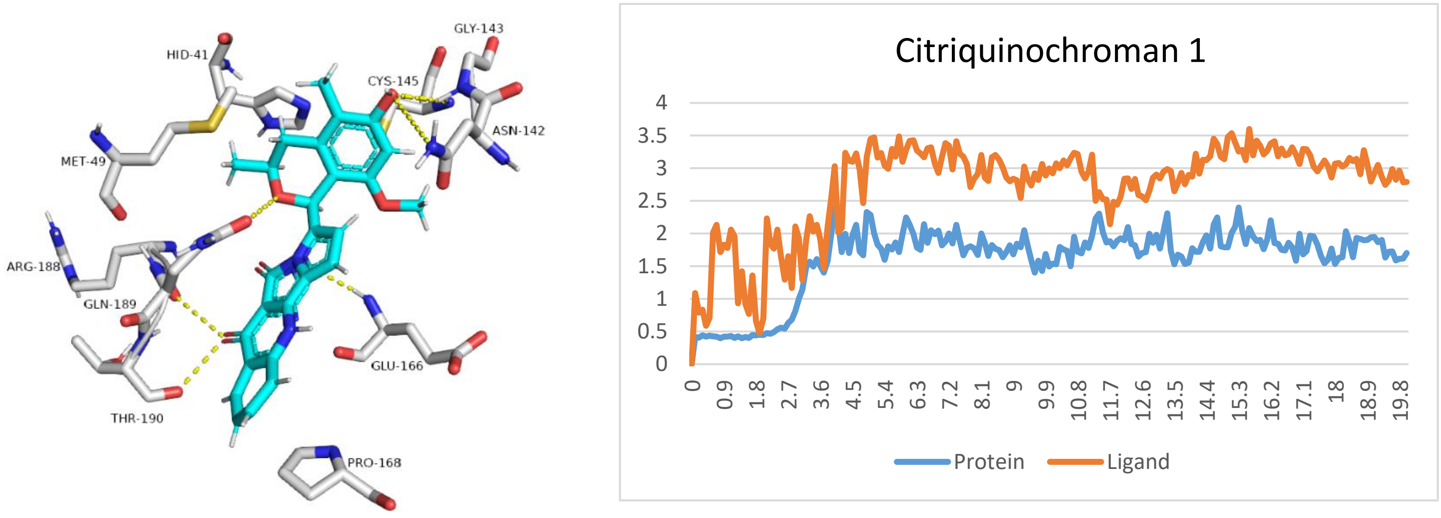

Figure S2. RMSDs (right) of the M^{pro} enzyme-ligand complex and the ligand (**1**). Docking binding pose of **1** (left) inside the binding pocket of the M^{pro} enzyme.

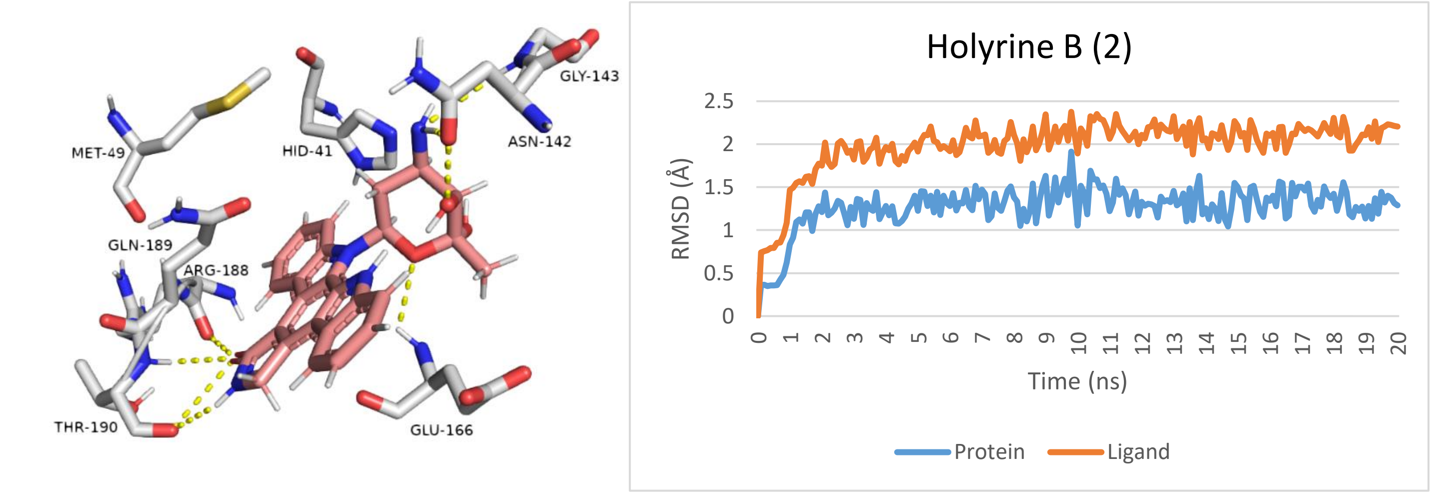

Figure S3. RMSDs (right) of the M^{pro} enzyme-ligand complex and the ligand (**2**). Docking binding pose of **2** (left) inside the binding pocket of the M^{pro} enzyme.

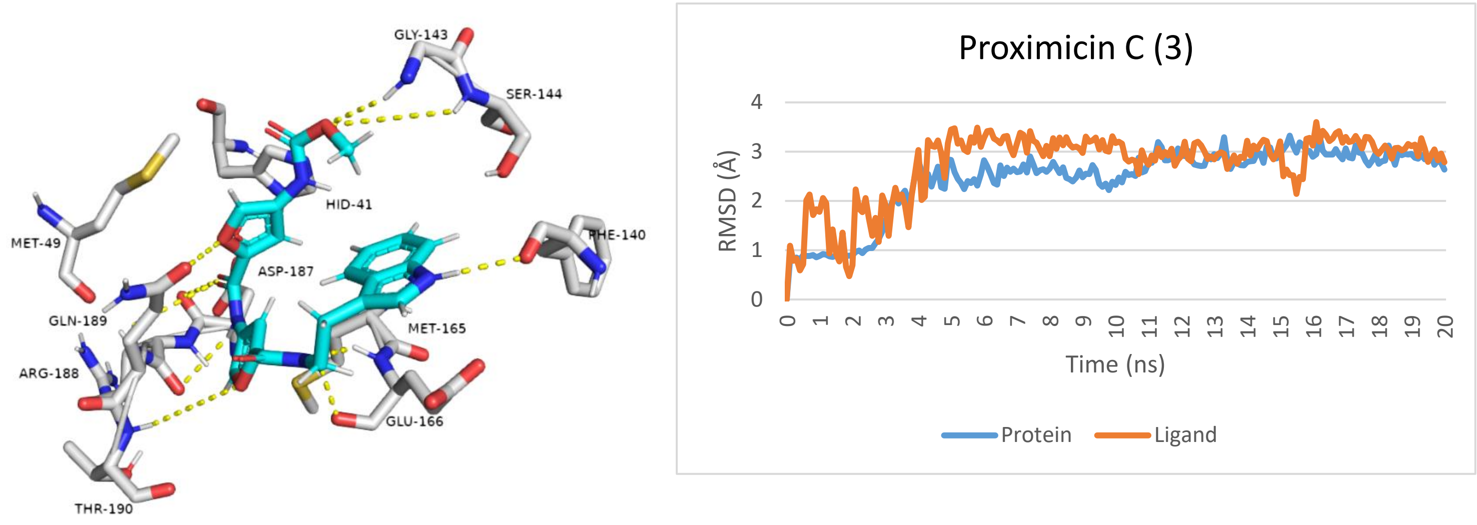

Figure S4. RMSDs (right) of the M^{pro} enzyme-ligand complex and the ligand (**3**). Docking binding pose of **3** (left) inside the binding pocket of the M^{pro} enzyme.

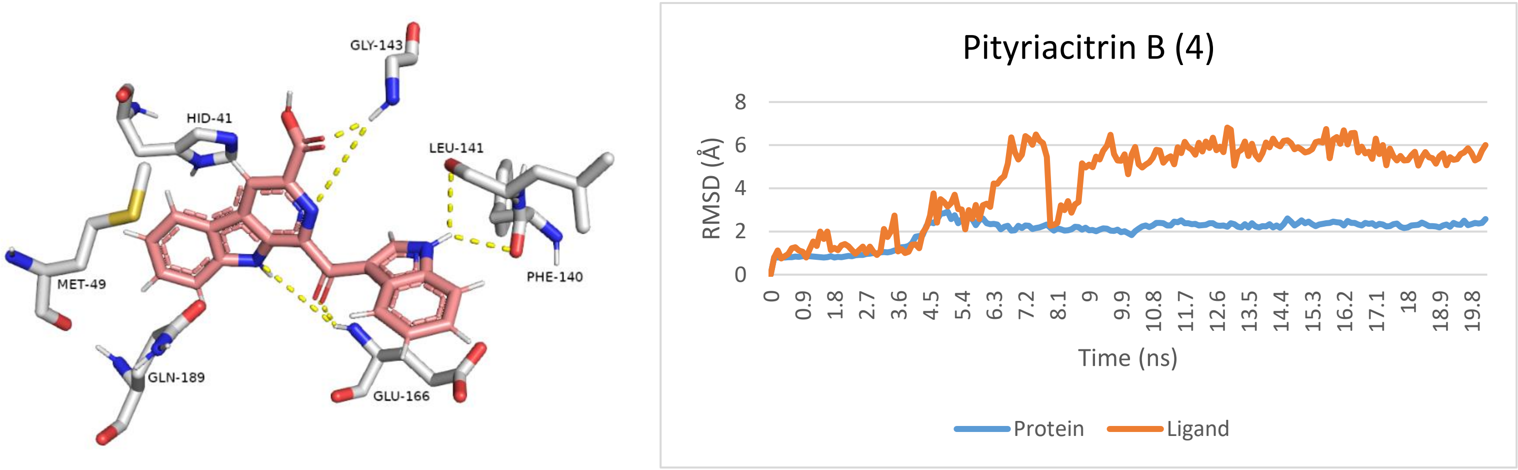

Figure S5. RMSDs (right) of the M^{pro} enzyme-ligand complex and the ligand (**4**). Docking binding pose of **4** (left) inside the binding pocket of the M^{pro} enzyme.

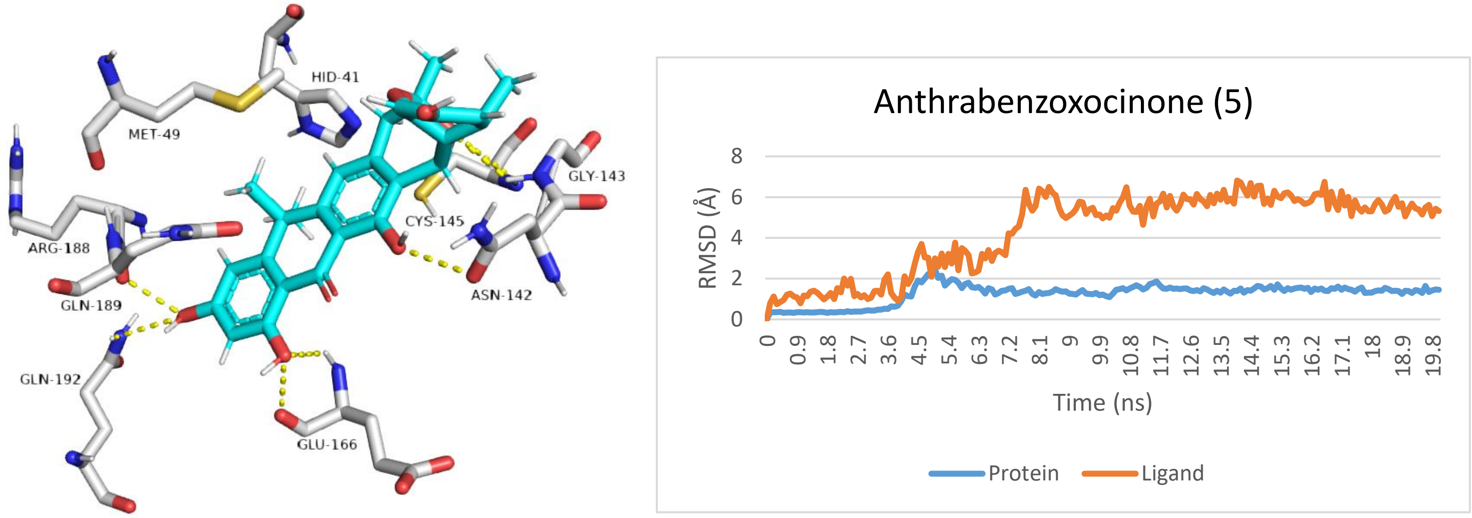

Figure S6. RMSDs (right) of the Mpro enzyme-ligand complex and the ligand (**5**). Docking binding pose of **5** (left) inside the binding pocket of the Mpro enzyme.

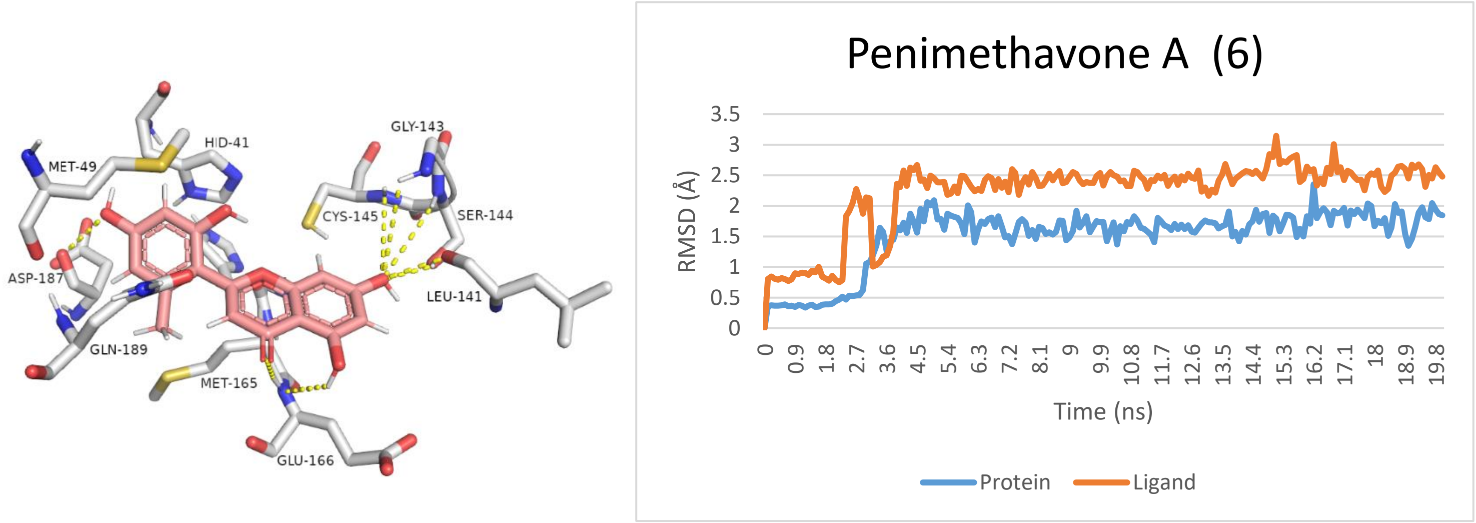

Figure S7. RMSDs (right) of the Mpro enzyme-ligand complex and the ligand (**6**). Docking binding pose of **6** (left) inside the binding pocket of the Mpro enzyme.

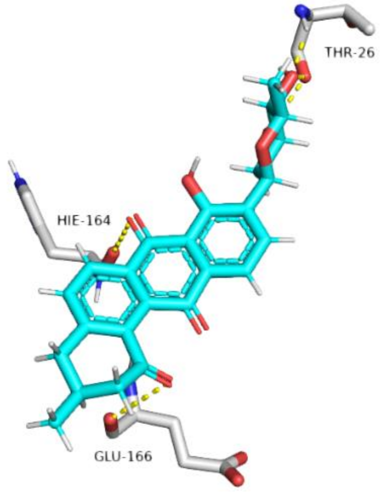

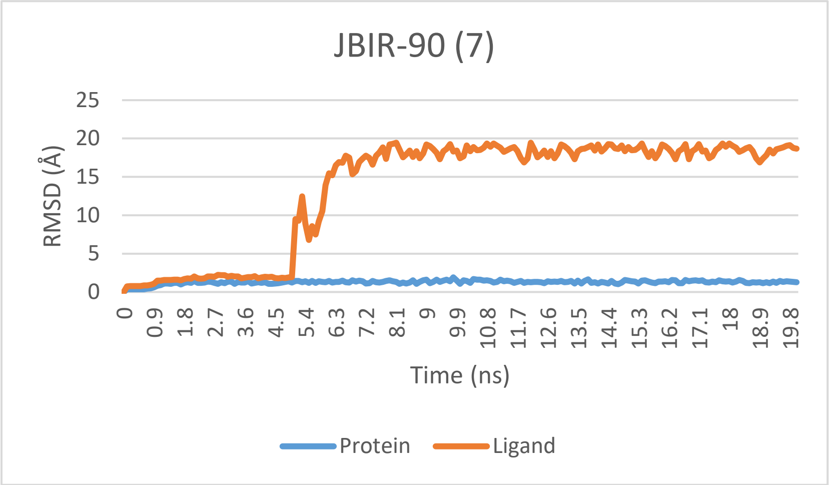

Figure S8. RMSDs (right) of the M^{pro} enzyme-ligand complex and the ligand (**7**). Docking binding pose of **7** (left) inside the binding pocket of the M^{pro} enzyme.

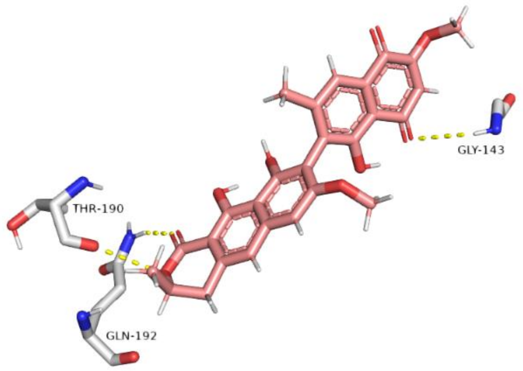

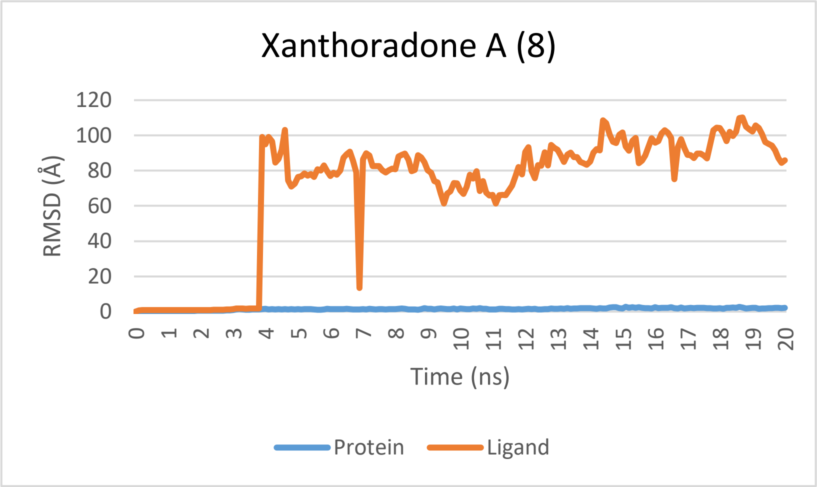

Figure S9. RMSDs (right) of the M^{pro} enzyme-ligand complex and the ligand (**8**). Docking binding pose of **8** (left) inside the binding pocket of the M^{pro} enzyme.

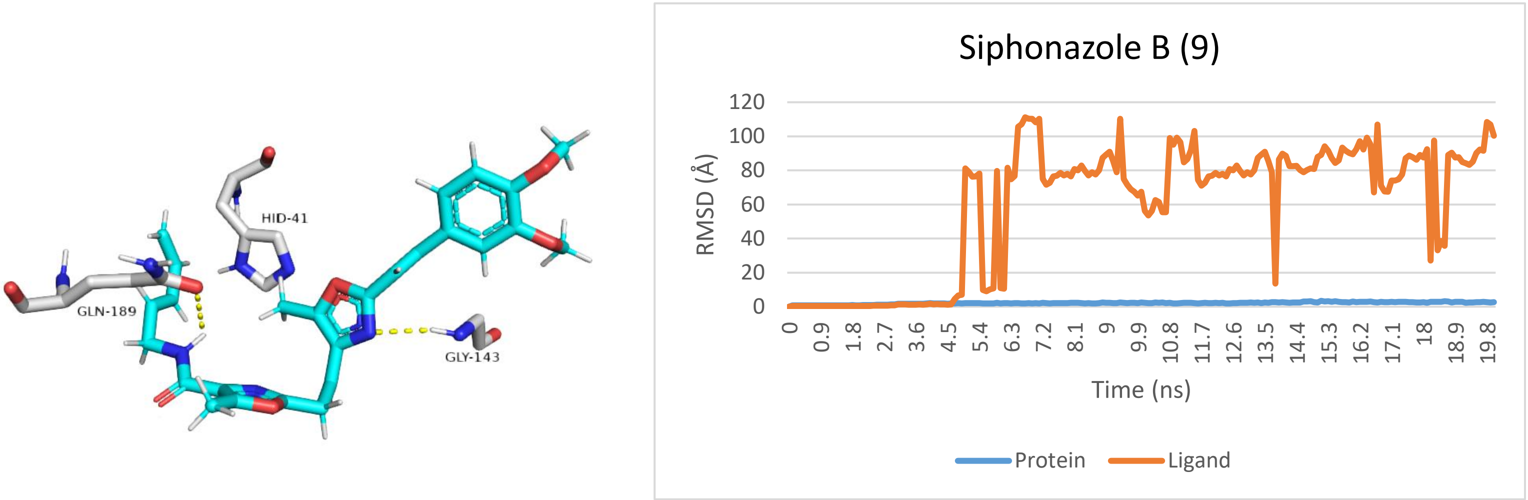

Figure S10. RMSDs (right) of the M^{pro} enzyme-ligand complex and the ligand (**9**). Docking binding pose of **9** (left) inside the binding pocket of the M^{pro} enzyme.

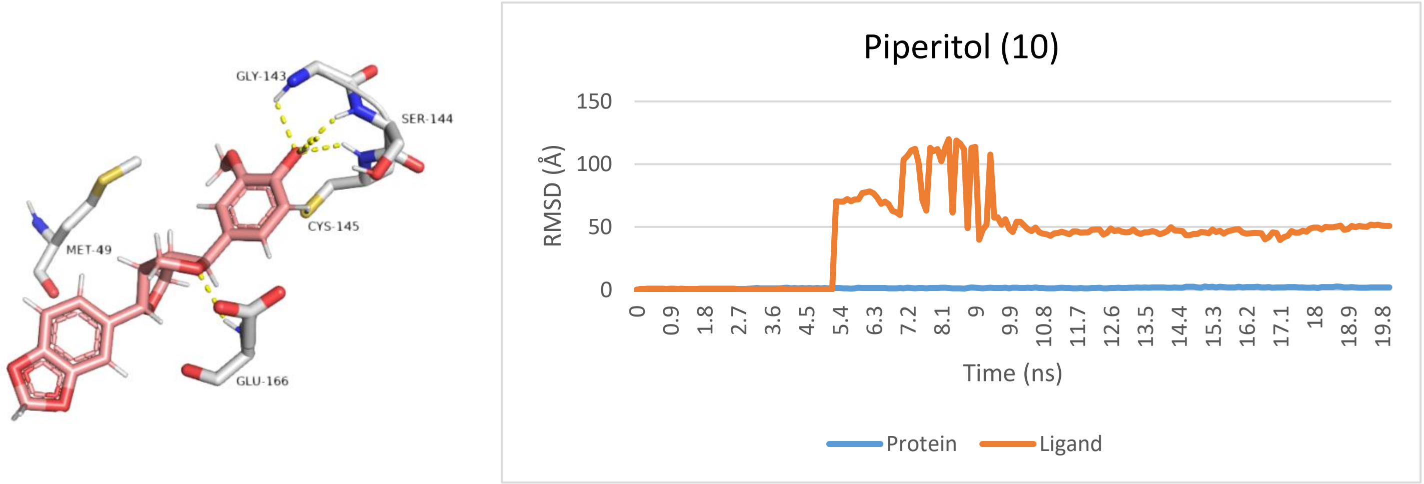

Figure S11. RMSDs (right) of the M^{pro} enzyme-ligand complex and the ligand (**10**). Docking binding pose of **10** (left) inside the binding pocket of the M^{pro} enzyme.

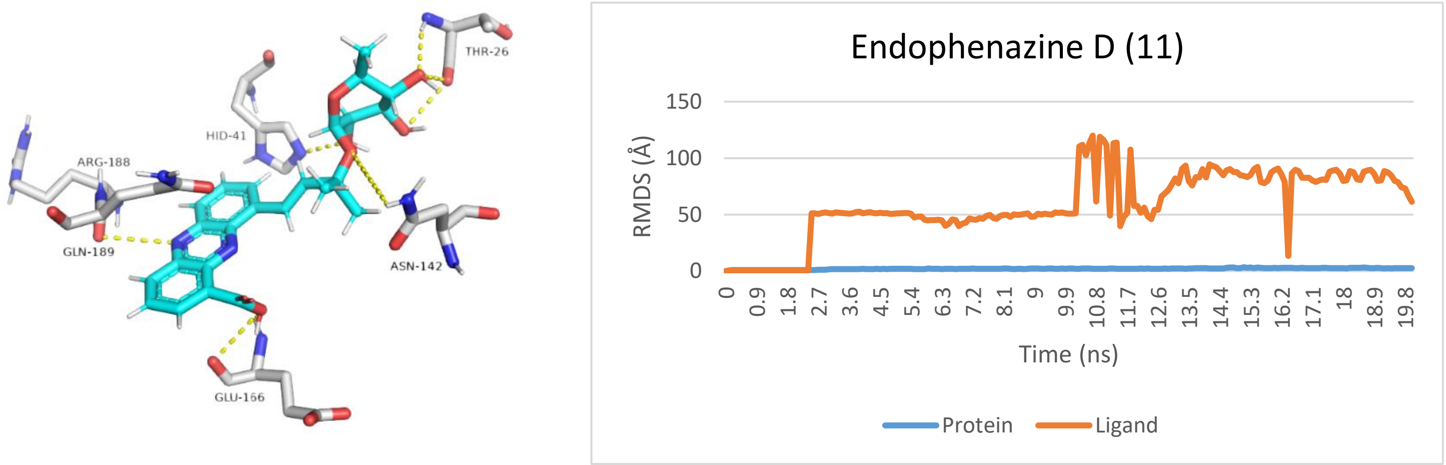

Figure S12. RMSDs (right) of the M^{pro} enzyme-ligand complex and the ligand (**11**). Docking binding pose of **11** (left) inside the binding pocket of the M^{pro} enzyme.

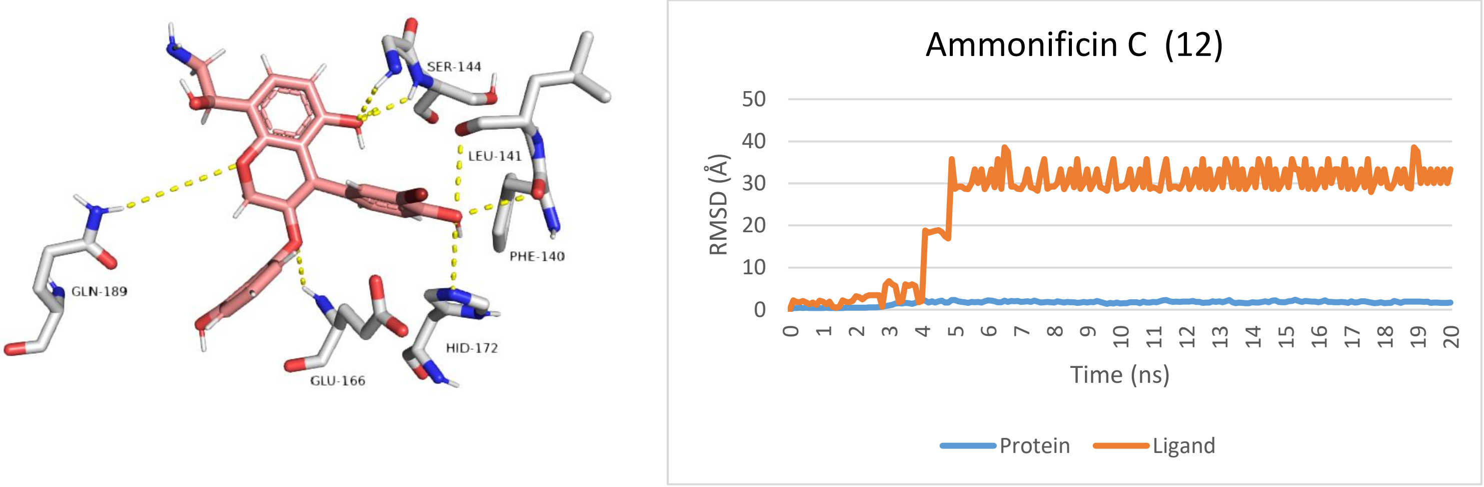

Figure S13. RMSDs (right) of the M^{pro} enzyme-ligand complex and the ligand (**12**). Docking binding pose of **12** (left) inside the binding pocket of the M^{pro} enzyme.
